# E-Cigarettes: A New Threat to Cardiovascular Health – A World Heart Federation Policy Brief

**DOI:** 10.5334/gh.1076

**Published:** 2021-10-18

**Authors:** Eduardo Bianco, Andrii Skipalskyi, Fastone Goma, Hanin Odeh, Koji Hasegawa, Mawya Al Zawawi, Michal Stoklosa, Regina Dalmau, E Ulysses Dorotheo, Florence Berteletti, Jeremiah Mwangi, Yunshu Wang

**Affiliations:** 1 Framework Convention Alliance, UY; 2 Advocacy Center LIFE, UA; 3 Eden University, ZM; 4 NCD Child Global Governing Council, JO; 5 Clinical Research Institute National Hospital Organization Kyoto Medical Center, JP; 6 Framework Convention Alliance Eastern Mediterranean Region, JO; 7 Institute for Health Research and Policy, University of Illinois Chicago, US; 8 University Hospital La Paz, Madrid, ES; 9 Southeast Asia Tobacco Control Alliance, PH; 10 World Heart Federation, CH

**Keywords:** Electronic Cigarette, E-Cigarette, Electronic Nicotine Delivery Systems, ENDS, Vaping, Cardiovascular Disease, CVD, Risk Factor, Cardiovascular Health, Public Health, Tobacco Control, Policy, Regulation

## Abstract

Tobacco is widely recognized as a leading cause of cardiovascular morbidity and mortality, accounting for approximately seventeen percent of all cardiovascular disease deaths globally. Electronic nicotine delivery systems such as e-cigarettes have been developed and advertised as safer alternatives to traditional tobacco cigarettes. Aggressive marketing strategies, as well as misleading claims by manufacturers, have largely contributed to the belief that e-cigarettes are harmless.

In reality, e-cigarettes are far from innocuous. E-cigarette solutions and aerosols generally contain harmful substances that are commonly found in tobacco cigarette emissions. A growing body of literature suggests that e-cigarettes are associated with an increased risk of cardiovascular morbidity and mortality. In addition, the effectiveness of e-cigarettes as smoking cessation tools has yet to be determined. Concerningly, most smokers do not give up on tobacco cigarettes and eventually become dual users.

Unregulated, e-cigarettes constitute a serious threat to established tobacco control policies. Fortunately, many countries have demonstrated that strong regulations were effective in protecting their populations from the dangers of e-cigarettes. The World Heart Federation recommends applying the precautionary principle and a set of measures to protect vulnerable populations, prevent exposure to second-hand smoking, and address misleading claims.

In this regard, we recommend that governments, policymakers, and other relevant stakeholders enact or support the following measures, among others:

- Prohibit the sale and distribution of e-cigarettes to minors, as well as the use of flavouring agents.
- Prohibit the use of e-cigarettes anywhere tobacco cigarettes have been banned.
- Prohibit marketing, advertising, and misleading claims regarding e-cigarettes.
- Apply excise taxes on e-cigarettes.
- Conduct more research regarding the long-term effects of e-cigarettes on cardiovascular health.

Lastly, countries that have banned the commercialization of e-cigarettes should maintain these measures.

## The tobacco epidemic and cardiovascular diseases

Cardiovascular diseases remain the leading cause of mortality worldwide and are responsible for over 18.6 million deaths annually [1,2].

Cigarette smoking affects nearly every organ in the human body and can result in the development of diseases such as cancers, pulmonary diseases, and cardiovascular diseases [3]. Tobacco is widely recognised as a major risk factor for cardiovascular morbidity and mortality, accounting for approximately 17% of all cardiovascular disease deaths globally [2,4].

## The rise in popularity of electronic cigarettes

Electronic nicotine delivery systems (ENDS) such as electronic cigarettes have been developed and marketed as a safer alternative to traditional tobacco cigarettes [5]. Since their introduction in 2004, the popularity of e-cigarettes has surged exponentially worldwide [6]. In 2018, e-cigarettes were sold in more than 102 countries and purchased by consumers of different ages [6–7]. At that time, the e-cigarette market was worth USD 11.26 billion and was projected to reach USD 26.84 billion by 2023 [8,9].

E-cigarettes have become particularly popular among young populations in recent years [10]. For instance, the use of e-cigarettes in US middle and high school students increased from 1.5% in 2011 to 20.8% in 2018 [11]. In 2016, e-cigarettes were far more popular than traditional cigarettes among US youth [12].

**Terminologies**

- Electronic nicotine delivery systems (ENDS) are commonly referred to as electronic cigarettes or e-cigarettes in the general public. Therefore, the terms ‘ENDS’ and ‘e-cigarette’ are used interchangeably in this paper.
- Electronic non-nicotine delivery systems (ENNDS), in contrast to ENDS, do not contain nicotine. Their effects on the cardiovascular system are not discussed in this paper.
- Heated tobacco products (HTP) are not a type of ENDS. HTPs create an aerosol by heating tobacco and should be considered and regulated, accordingly, as tobacco products [13].

### Evolution of e-cigarettes

The design of ENDS is changing rapidly, and products are constantly enhanced to satisfy consumer demands [6]. In fact, the word ‘e-cigarette’ designates a group of products that differ considerably in terms of shapes, capabilities, and functionalities [14].

First-generation e-cigarettes, also called *ciga-likes*, were designed to resemble traditional cigarettes and are characterized by a low-voltage battery [8,15]. Second-generation e-cigarettes are larger, shaped like a pen, and are equipped with a refillable reservoir, as well as a rechargeable battery [8,15]. Third-generation e-cigarettes, sometimes also referred to as mods, diverge from previous generations in terms of both shape and size. These e-cigarettes are defined by large refillable tanks and batteries with adjustable power outputs [8,15]. Higher voltages result in higher temperatures and lead to an increased delivery of nicotine [5]. Modern e-cigarettes, such as pods, have a high-tech design and have adopted prefilled cartridges that contain concentrations of nicotine comparable to that in traditional cigarettes, as well as a variety of flavours [8,10].

Regardless of the model, all ENDS deliver nicotine by heating and transforming a solution, known as e-liquid, into an aerosol inhaled by the user [5]. E-cigarettes are generally equipped with a mouthpiece, a sensor, a battery, a reservoir/tank, and a heating element/atomiser [10]. Inhaling through the mouthpiece triggers the sensor and activates the heating element. The latter then heats and vaporizes the e-liquid into an aerosol [10,15].

### Marketing and appeal of e-cigarettes

Electronic cigarettes are regularly advertised as harm reduction tools and smoking cessation aids [7]. Aggressive marketing strategies, as well as misleading claims by manufacturers, have largely contributed to the belief that e-cigarettes are harmless or less harmful than conventional cigarettes [15,16].

Therefore, e-cigarettes are generally perceived as safer, healthier, and less addictive substitutes for traditional tobacco cigarettes [5,8,15]. In addition, e-cigarettes are frequently employed as a smoking cessation tool and/or as a way to circumvent tobacco control policies [5,15]. Lastly, the novelty of the product, the polished design, and the immense variety of flavours make e-cigarettes particularly appealing to youth [10].

### Health concerns regarding e-cigarettes

A growing body of literature suggests that e-cigarettes are associated with an increased risk of cardiovascular and other morbidity and mortality [3,17]. A number of studies have confirmed that e-cigarettes are far from harmless and that e-liquids and e-cigarette aerosols contain harmful substances that are commonly found in conventional cigarette emissions [10,17]. Albeit present at substantially lower levels, these components could potentially have similar adverse effects on the cardiovascular system, and electronic cigarettes should not be considered safe until proven otherwise [10,17].

In the present case, the precautionary principle should be applied.

## Electronic cigarette constituents

While e-cigarettes do not contain or involve the combustion of tobacco, they still generate toxic substances that are typically found in traditional cigarette smoke [5,18]. These constituents are generally present at a significantly lower level and vary considerably depending on the device and the e-liquid [12,14]. The additive or synergistic nature of these components is currently unknown [11].

### E-liquids

E-liquids are usually a mixture of propylene glycol, glycerine, nicotine, and flavouring agents [11]. Propylene glycol and glycerine, both regarded as safe for oral consumption, can transform into harmful substances when exposed to high temperatures [6,11]. Flavouring agents largely contribute to the massive variation in e-liquid and aerosol composition and may even contain harmful substances not present in traditional cigarettes [8]. While the vast majority of flavouring agents are considered safe for ingestion, data regarding their safety when inhaled is limited [11,12]. In fact, some flavouring agents such as cinnamaldehydes are known to be toxic when inhaled [11].

### E-cigarette aerosols

The composition of the aerosol depends on the devices and e-liquids [11]. Components such as nicotine, carbonyl compounds, particulate matter, metals, volatile organic compounds, and tobacco-specific nitrosamines have been routinely detected in e-cigarette emissions [6,11,15]. The presence of these substances could be a threat to the health of e-cigarette users and bystanders.

### Nicotine

Modern e-cigarettes can deliver concentrations of nicotine that are comparable to traditional cigarettes [8]. Nicotine is directly associated with the development of cardiovascular diseases and is responsible for an increase in heart rate, blood pressure, and vasoconstriction [6]. Studies have also demonstrated that this substance can induce inflammation, endothelial dysfunction, angiogenesis, lipogenesis, and atherosclerosis [6,11,15].

Nicotine is widely recognized as a highly addictive drug, and concentrations delivered by modern e-cigarettes are able to both generate and maintain dependence [12,15]. Nicotine can also affect brain development and can be especially hazardous for infants, adolescents, and pregnant women [6,8].

### Carbonyl compounds

Carbonyl compounds such as acetaldehyde, formaldehyde, and acrolein are produced during the thermal degradation of propylene glycol and glycerine [15]. The cardiovascular effects of these aldehydes include oxidative stress and inflammation [11,15]. Acrolein is also known to induce endothelial dysfunction, vascular injury, platelet activation, and atherosclerosis [6,11]. Modern e-cigarettes are capable of producing similar levels of carbonyl compounds as those produced by tobacco cigarettes [15].

### Particulate matter

Fine (PM2.5) and ultrafine (PM0.1) particulate matter are particles (< 2.5 µm and < 0.1 µm diameter, respectively) that can reach the general circulation through the lungs [11,15]. These particles are associated with the development of cardiovascular diseases and can be found at levels comparable to those of conventional cigarettes [11,15].

Exposure to PM2.5 can lead to the development of hypertension, oxidative stress, endothelial dysfunction, atherosclerosis, arrhythmia, coronary artery disease, and myocardial infarction [11,15]. PM2.5 is also present in second-hand smoke exhaled by e-cigarette smokers and could be of particular importance in terms of public health because even low levels of exposure could cause harm [15].

### Heavy metals

Various types and concentrations of metals have been detected in different studies [6,11]. Owing to this variance, their role in the development of cardiovascular diseases is still being assessed.

### Second-hand smoking

E-cigarette users demonstrably exhale aerosols containing nicotine and other harmful constituents in the air, even if electronic cigarettes do not emit side-stream smoke [5]. There is strong evidence supporting the idea that e-cigarettes can affect the quality of air indoors and that bystanders breathe in aerosols and their constituents in some capacity [5,15].

## Cardiovascular effects of electronic cigarettes

Many studies have confirmed that e-cigarettes are associated with an increased risk of cardiovascular morbidity and mortality [3,17]. The use of e-cigarettes is irrefutably correlated with physiological changes that are typically observed in individuals who develop cardiovascular diseases [5].

### Haemodynamic changes

E-cigarette use is associated with an increase in heart rate and blood pressure, as well as with an unnatural heart rate variability pattern [11,15,19]. These effects could be attributed to nicotine, as they are generally not observed in people using nicotine-free e-liquids [11]. Furthermore, sustained use of e-cigarettes can lead to permanent elevation in heart rate and blood pressure [11].

### Vascular changes

Electronic cigarettes are associated with inflammation, oxidative stress, endothelial dysfunction, vascular injury, arterial stiffness, and atherosclerosis [11,15,19]. In the long term, e-cigarettes can also lead to elevated levels of oxidative stress [11].

### Platelet changes

The use of e-cigarettes is associated with an increase in platelet aggregation, although most studies on platelets have been conducted *in vitro* or in animals [8].

### Risk of myocardial infarction

E-cigarettes are associated with an increased risk of myocardial infarction, and habitual e-cigarette users are 1.79 times more likely to develop myocardial infarction than non-smokers [11]. By contrast, chronic tobacco cigarette smokers are 2.72 times more likely to experience myocardial infarction than non-smokers [19].

## Smoking cessation and initiation

### Potential as a smoking cessation tool

Electronic cigarettes are often marketed as effective smoking cessation tools. However, they have never been officially approved for this purpose [10]. In fact, multiple sources have indicated that e-cigarette users are less likely to quit smoking [5,16].

A number of studies have suggested that e-cigarettes could be more effective than nicotine replacement therapies (NRT) and as effective as varenicline for the cessation of traditional cigarettes when all interventions benefit from behavioural support [8,19]. However, recent data have challenged these findings [20]. Moreover, a significant number of ‘quitters’ became chronic e-cigarette smokers because of the addictive nature of nicotine [19].

E-cigarettes were initially developed as a safer alternative to tobacco cigarettes and were never meant to become smoking cessation tools. E-cigarettes are neither officially approved for smoking cessation therapies nor are pharmacologically controlled [18]. There is no standard dosage or dosing regimen for smoking cessation [16]. In comparison, NRTs are used for only short periods of time, and fixed doses are administered through oral or transdermal routes [18,19]. In addition, NRTs are not associated with an increased risk of cardiovascular diseases [18].

### Potential as a smoking reduction tool

Data suggest that a large majority of e-cigarette users do not give up tobacco cigarettes and become dual users [5]. Most dual users utilize e-cigarettes as a supplement to traditional cigarettes and are generally exposed to a higher number and concentration of harmful constituents [8]. As a result, dual users may incur higher risks of developing cardiovascular diseases.

Some dual users employ electronic cigarettes as a smoking reduction tool by replacing a number of tobacco cigarettes with e-cigarettes [8]. Unfortunately, the dose-response relationship between tobacco smoking and the risk of cardiovascular diseases is nonlinear [17]. Consequently, the decrease in risk will not be proportional to the number of substituted cigarettes, and even low levels of exposure will lead to a significant increase in the risk of cardiovascular diseases [15,21].

### Potential as a harm reduction tool

Although not harmless, current evidence suggests that completely switching from tobacco cigarettes to electronic cigarettes would reduce exposure to toxicants in terms of quality and quantity, and could result in health improvements in the short term [8,12]. Nonetheless, surveys indicate that a majority of e-cigarette users are dual users of both conventional and electronic cigarettes, which carries an increased health risk [5].

### Potential for smoking initiation and relapse

Even with irregular exposure, youth are still extremely vulnerable to the effects of nicotine [10]. The use of e-cigarettes in young populations is generally associated with a transition to traditional tobacco cigarettes [10]. Studies suggest that adolescents who smoke e-cigarettes are 3.5 times more prone to start smoking conventional cigarettes than non-smoking peers [10]. This transition could be explained by their addiction to nicotine and a set of acquired behavioural and social habits [22]. Moreover, there are also some concerns regarding whether e-cigarettes could incite ex-smokers to re-start smoking [8].

## Limitations of studies on electronic cigarettes

The extensive number of e-cigarette devices and e-liquids will inevitably lead to different study outcomes [12]. Different devices and solutions produce drastically different aerosols, in terms of both composition and concentration [12]. Furthermore, the endless range of flavouring agents adds several layers of complexity to the problem [12]. The profile of the study population, as well as the consumption technique, will also directly influence the results [11].

Consequently, confirming the precise health effects of e-cigarettes and extrapolating the study results to the general population is a challenging task. Research protocols, methods, and designs need to be standardized and improved [12]. Additionally, the paucity of data on the long-term effects of e-cigarettes is a particularly pressing issue [8].

## Regulation of electronic cigarettes

The regulation of e-cigarettes can vary significantly from one country or region to another, with no global standard currently available [6]. Governments generally categorize e-cigarettes as tobacco products, imitation tobacco products, medicinal products, pharmaceutical products, consumer products, poisons, or electronic nicotine delivery systems [9]. The policies and regulations that can be applied to e-cigarettes depend heavily on their classification [9]. Some nations have chosen to enact new legislation to regulate e-cigarettes, while others have opted to apply or amend existing laws [9]. In 2018, electronic cigarettes were banned in 40 countries worldwide, while being entirely unregulated in 39 other countries [7,8]. Generally, countries have opted to prohibit or restrict possession, use, marketing, and flavours of e-cigarettes [8].

E-cigarettes are an extremely diverse group of products with more than 15,500 different types of flavours [11,23]. This diversity perfectly illustrates why the regulation of e-cigarettes is necessary. Furthermore, e-cigarettes are rapidly and constantly being replaced by newer models, and regulations need to be responsive to match this unrelenting pace.

### The tobacco industry

In 2019, the Convention Secretariat of the WHO Framework Convention on Tobacco Control (FCTC) reiterated that there is a ‘*fundamental and irreconcilable conflict between the tobacco industry’s interests and public health policy interests in accordance with Article 5.3 of the Convention and its guidelines*’ [7].

All prominent transnational tobacco corporations are currently involved in e-cigarette businesses [5]. Following their signature modus operandi, these companies have become highly influential in the policymaking of e-cigarettes and have positioned themselves at multiple levels of the decision-making process [5]. At the academic level, the tobacco industry has funded several studies that severely downplayed the dangers of e-cigarettes [8].

### Marketing strategies

The marketing of e-cigarettes plays a central role in shaping the opinions of the general public [16]. The tobacco industry has a long history of using deceitful and misleading claims to advertise its products [10]. One of its objectives is to ensure that e-cigarettes are perceived as safer or harmless alternatives to conventional tobacco cigarettes [5]. In addition, research data suggest that youth who have been exposed to such marketing strategies were more prone to smoke e-cigarettes [10].

### Threats to tobacco control laws

Undeniably, the tobacco industry is capitalizing on electronic cigarettes to interfere with existing tobacco control laws, and is pushing for e-cigarette-friendly regulation while continuing to oppose effective regulation of conventional cigarettes and other tobacco products [7]. Tobacco companies are exploiting loopholes in current legislation to sabotage tobacco denormalization and encourage defiance against tobacco control policies [7,14]. For instance, in the absence of regulation, e-cigarettes can and will be used to circumvent smoke-free policies [7].

### Regulation of e-cigarettes in Canada

The Tobacco and Vaping Products Act prohibits the sale and distribution of e-cigarettes to minors, as well as the marketing and advertising of e-cigarettes to young populations [24,25]. The legislation also requires manufacturers to include appropriate labelling and health warnings on the packages of e-cigarettes [24].

Prohibiting the sale and distribution of e-cigarettes to minors has considerably reduced the uptake of e-cigarettes among young Canadians [25]. Moreover, young people demonstrated a better understanding of the potential dangers of e-cigarettes and reported a decrease in accessibility [25]. Evidence also suggests that populations residing in countries with stricter marketing regulations are generally less exposed to e-cigarette advertisements [26].

### Regulation of e-cigarettes in Finland

The Tobacco Act 2016 prohibits the sale and distribution of e-cigarettes to minors, online sales of e-cigarettes, flavouring agents that ‘*smell or taste other than one of tobacco*’ [27], and the use of e-cigarette anywhere tobacco cigarettes have been banned [27,28]. The legislation also prohibits the marketing and advertising of e-cigarettes, as well as the use of misleading claims [27–28]. Moreover, manufacturers are required to include appropriate labelling and health warnings on e-cigarette packages [28].

The Tobacco Act 2016 also subjects e-cigarette solutions to excise duty [27]. A number of studies have demonstrated that an increase in e-cigarette prices is directly related to a decline in e-cigarette sales [29,30]. Therefore, the application of an excise tax should increase revenue for governments and decrease the use of e-cigarettes in the population. Policymakers should also consider the fact that e-cigarettes are substitutes for traditional cigarettes [29,30].

The National Supervisory Authority for Welfare and Health (Valvira) oversees compliance with established regulations [27]. Manufacturers and importers must notify Valvira before introducing a new product in the market [27]. Additionally, grocery stores, kiosks, and specialised shops must acquire a license to sell e-cigarettes [27,28].

Finland has adopted a relatively strong stance on e-cigarettes, and the number of users is presently lower than in other European countries [27]. Nonetheless, a number of infringements and legal cases have been recorded during the past few years, and continued monitoring remains essential [27].

## Conclusion

In recent years, electronic cigarettes have become immensely popular, especially among youth. The latest data indicate that these products are far from harmless and produce harmful aerosol components that are typically found in traditional cigarette smoke.

E-cigarettes are associated with an increased risk of cardiovascular morbidity and mortality. Their effects on the cardiovascular system could be substantial. Nevertheless, more studies on their long-term effects are needed, and addressing this gap in knowledge should be a priority [11]. When assessing the public health consequences of e-cigarettes, other diseases such as cancers and pulmonary diseases should also be considered.

Evidence suggests that tobacco smokers may benefit from completely switching from traditional cigarettes to e-cigarettes. However, to date, e-cigarettes have demonstrated limited efficacy as a smoking cessation or reduction tool, and dual use is common. Ironically, these products can be highly addictive and can initiate tobacco cigarette smoking.

The regulation of electronic cigarettes is of paramount importance and should focus on protecting both users and bystanders while preventing initiation and uptake. E-cigarettes are changing rapidly, and regulations should ensure safety and quality standards.

Finally, future legislation on electronic cigarettes should strengthen existing tobacco control laws. Countries that have banned the commercialization of e-cigarettes, based on the precautionary principle, should maintain these measures.

## Recommendations

The World Heart Federation recommends the following measures regarding research and studies on the cardiovascular effects of electronic cigarettes:

**Table:** 

Recommendations	Reasons

Conduct more research regarding the long-term effects of e-cigarettes on cardiovascular health.	To close the knowledge gap regarding the long-term effects of e-cigarettes on the cardiovascular system.
Medical and scientific journals should desist from publishing, and presenting at congress, studies funded by the tobacco industry.	To prevent biased study results. To prevent conflicts of interest. To ensure transparency.

The World Heart Federation recommends the following measures to prevent the initiation and uptake of electronic cigarettes by non-smokers and young populations:

**Table:** 

Recommendations	Reasons

Prohibit the sale and distribution of e-cigarettes to minors.	To bring to an end the use of e-cigarettes by minors.
Prohibit flavouring agents.	To make e-cigarettes less appealing to young populations. To prevent the use of constituents that can be potentially harmful and unsafe for inhalation. To facilitate the regulation of e-cigarette solutions.
Prohibit online sales of e-cigarettes.	To make e-cigarettes less accessible to minors. To prohibits sales of unregulated products.
Require labelling and health warning on the packages of e-cigarettes.	To warn consumers about the potential dangers of e-cigarettes. To replicate a proven strategy used against tobacco cigarettes. To ensure child-resistant packaging.
Apply excise taxes on e-cigarettes.	To make e-cigarettes unaffordable to youth. To raise income for governments.
Educate teachers and parents.	To address the e-cigarette epidemic in middle and high schools.

The World Heart Federation recommends the following measure to protect the general population from the second-hand smoking of electronic cigarettes:

**Table:** 

Recommendations	Reasons

Prohibit the use of e-cigarettes anywhere tobacco cigarettes have been banned.	To reinforce smoke-free legislations in both public places and indoors.

The World Heart Federation recommends the following measures to address aggressive marketing strategies and misleading claims regarding electronic cigarettes:

**Table:** 

Recommendations	Reasons

Prohibit marketing, advertising, and misleading claims regarding e-cigarettes.	To reduce exposure to aggressive marketing strategies and advertisements. To replicate a proven strategy used against tobacco cigarettes. To prevent misleading claims that could encourage consumers to use a potentially harmful product. To prevent misleading claims regarding the innocuousness of e-cigarettes. To prevent misleading claims regarding the addictive nature of e-cigarettes. To prevent misleading claims regarding the effectiveness of e-cigarettes as smoking cessation tools.
Continue to monitor marketing, advertising, and misleading claims regarding e-cigarettes.	To reinforce the prohibition law. To ensure regulatory compliance. To minimize and prevent interferences from the tobacco industry with regard to marketing and advertising.
